# The potential future contribution of shipping to acidification of the Baltic Sea

**DOI:** 10.1007/s13280-017-0950-6

**Published:** 2017-10-05

**Authors:** David R. Turner, Moa Edman, Julián Alberto Gallego-Urrea, Björn Claremar, Ida-Maja Hassellöv, Anders Omstedt, Anna Rutgersson

**Affiliations:** 1 Department of Marine Sciences, University of Gothenburg, Box 461, 405 30 Gothenburg, Sweden; 2 SMHI, Sven Källfelts gata 15, 426 71 Västra Frölunda, Sweden; 3 Department of Earth Sciences, Uppsala University, Villavägen 16, 752 36 Uppsala, Sweden; 4 Department of Mechanics and Maritime Sciences, Chalmers University of Technology, 412 96 Gothenburg, Sweden

**Keywords:** Acidification, Baltic Sea, Biogeochemical modelling, Shipping, Scrubbers

## Abstract

International regulation of the emission of acidic sulphur and nitrogen oxides from commercial shipping has focused on the risks to human health, with little attention paid to the consequences for the marine environment. The introduction of stricter regulations in northern Europe has led to substantial investment in scrubbers that absorb the sulphur oxides in a counterflow of seawater. This paper examines the consequences of smokestack and scrubber release of acidic oxides in the Baltic Sea according to a range of scenarios for the coming decades. While shipping is projected to become a major source of strong acid deposition to the Baltic Sea by 2050, the long-term effect on the pH and alkalinity is projected to be significantly smaller than estimated from previous scoping studies. A significant contribution to this difference is the efficient export of surface water acidification to the North Sea on a timescale of 15–20 years.

## Introduction

### Ocean acidification

It is now well-established that the increasing concentration of carbon dioxide in the atmosphere is causing a continuing reduction in the pH of surface ocean water at a current rate close to 0.002 pH units per year (Rhein et al. 2013); the accumulated decrease since the onset of industrialisation is estimated to be 0.1 pH units. Carbon dioxide is, however, not the only anthropogenic acid that has the potential to reduce oceanic pH. High temperature combustion leads to the formation of nitrogen oxides (NO_X_), while combustion of sulphur-rich fuels releases a mixture of sulphur oxides (SO_X_) to the atmosphere. In the atmosphere, these oxides are converted to nitric and sulphuric acids, respectively.

The chemical consequences of CO_2_ uptake and strong acid deposition are very different. Uptake of carbon dioxide causes a change in the relative proportions of dissolved carbon dioxide, carbonate ions, and bicarbonate ions in the water, but the total alkalinity is not affected. This uptake process is reversible should the partial pressure of CO_2_ decrease:

$$ {\text{CO}}_{ 2} \left( {\text{aq}} \right)  + {\text{ CO}}_{ 3}^{ 2- } \left( {\text{aq}} \right) \, \leftrightarrow {\text{ 2HCO}}_{ 3}^{ - } \left( {\text{aq}} \right) $$

Deposition of strong acid also causes a change in the relative proportions of the inorganic carbon species but, in contrast to CO_2_ uptake, is not reversible without the addition of a strong base:

$$ {\text{H}}^{ + } \left( {\text{aq}} \right)  + {\text{ CO}}_{ 3}^{ 2- } \left( {\text{aq}} \right) \, \to {\text{ HCO}}_{ 3}^{ - } \left( {\text{aq}} \right) $$

$$ {\text{H}}^{ + } \left( {\text{aq}} \right)  + {\text{ HCO}}_{ 3}^{ - } \left( {\text{aq}} \right) \, \to {\text{CO}}_{ 2} \left( {\text{aq}} \right)  + {\text{ H}}_{ 2} {\text{O}} $$

The research agenda in respect of these anthropogenic gas emissions has focused first on the atmosphere, and only later on the consequences for seawater. For many years, studies of the large-scale emission of anthropogenic CO_2_ had a clear focus on the greenhouse effect and climate change; the ocean featured only as a sink for a significant proportion of the anthropogenic CO_2_ (Solomon et al. 2007). In due course, the realisation that this oceanic uptake of CO_2_ causes a reduction in pH led to Ocean Acidification being dubbed “The Other CO_2_ Problem” (Doney et al. 2009); it is now included in the IPCC assessments of climate change (Rhein et al. 2013).

### Acidification by sulphur and nitrogen oxides

While the consequences of CO_2_ emissions are long-term and global, the consequences of SO_X_ and NO_X_ emissions have a more direct impact on air quality and human health. They are also more local since these oxides have a much shorter residence time than CO_2_, of the order of days (Rodhe et al. 2002), so that the greater part of the resulting acidic aerosols is deposited within tens or hundreds of kilometres of the source. Since the effects of SO_X_ and NO_X_ emissions are relatively local, and have consequences for human health, the first priority was to reduce the emissions of these gases from terrestrial activities, a process that continues to this day.

There have been few studies of ocean acidification by sulphuric and nitric acids derived from anthropogenic emissions of SO_X_ and NO_X_. Doney et al. (2007) carried out a global assessment using data from the 1990’s, which give a deposition flux equivalent to 4 Tmol protons per year after nitrification of deposited ammonia, compared with a CO_2_ uptake of 138 Tmol per year. These authors concluded that the resulting changes in alkalinity and dissolved inorganic carbon served to minimise the resulting decrease in surface water pH to less than 0.0001 pH units per year over most of the ocean, compared with a decrease of 0.002 pH units per year due to CO_2_ uptake (Orr 2011).

### Shipping as a source of ocean acidification

Hunter et al. (2011) modelled strong acid acidification in three sea areas on an annual basis, giving decreases of 0.00056, 0.00010 and 0.00027 pH units per year for the North Sea, Baltic Sea and South China Sea, respectively. Hassellöv et al. (2013) modelled the shipping-derived pH decreases worldwide on a seasonal basis, indicating that sea areas with heavy shipping traffic and seasonal stratification can be subject to larger pH decreases on a seasonal basis, although over a full year, the results were broadly compatible with the earlier work of Doney et al. (2007) and Hunter et al. (2011). Hagens et al. (2014) showed that the modelled pH decreases can be substantially modified by taking account of the rates of nitrification and of air-sea exchange of CO_2_. Omstedt et al. (2015) applied a process-oriented Baltic Sea model indicating that acidification due to the atmospheric deposition of acids peaked around 1980, with a cumulative pH decrease of approximately 0.01 in surface waters. The acid contribution of shipping was estimated to one order of magnitude less than that of land emissions. More recently, Stips et al. (2016) have used a spatially-resolved model to examine the potential impact of scrubber operation on acidification of the North Sea over a 1-year period. These authors conclude that the largest effects are confined to near-coastal areas, most particularly in the vicinity of major ports, where the acidifying effect due to SO_X_ can equal or exceed that due to CO_2_.

### Regulation of acid emissions from shipping

Since commercial shipping also acts as a source of these acidic oxides, emission regulations for shipping are under development, albeit at a much slower pace. A brief summary is given here; a more detailed account can be found in Turner et al. (2017). Environmental regulation of shipping activities worldwide is a responsibility of the International Maritime Organisation (IMO), an agency of the United Nations. Regulations are adopted in the framework of the International Convention for the Prevention of Pollution from Ships (MARPOL), where Annex VI is concerned with air pollution.

For SO_X_, this Annex limits the maximum sulphur content of marine fuel to 3.5%, due to be reduced to 0.5% in 2020. However, separate regulations apply in Sulphur Environmental Control Areas (SECA), where the limit is 0.1% from January 2015. These restrictions are relatively modest compared with those applying to terrestrial fuels: in the European Union, for example, the maximum sulphur content of fuels for terrestrial transport is 10 ppm (0.001%). At present the only SECA in European waters covers the Baltic Sea and the North Sea.

For NO_X_, the regulations are more complex since the maximum allowed emission depends on the age of the ship, in contrast to SO_X_ regulations that apply to all ships irrespective of age. The most stringent regulations apply to newly built ships within Nitrogen Environmental Control Areas (NECA). There is at present only one NECA, along the east coast of North America. A second NECA in the Baltic Sea is planned to come into effect in 2021 (HELCOM 2016).

### Scrubber technology

Although MARPOL Annex VI prescribes the maximum sulphur content of marine fuels in different areas, it explicitly allows the adoption of engineering solutions that achieve the same low flux of SO_X_ to the atmosphere. This has led to a strong interest in scrubber technology as a cost-effective method to meet the stricter emission regulations applying in SECA areas from 1 January 2015. The recent decision of IMO to reduce the maximum sulphur content of marine fuel from 2.5 to 0.5% worldwide from 2020 rather than 2025 can be expected to generate a more widespread interest in scrubber technology. Interestingly, while the regulations for atmospheric emission of SO_X_ are mandatory, there are no mandatory regulations concerning the properties of the scrubber effluents. Instead, IMO has issued guidelines that are suggested as a basis for national discharge regulations. The guidelines state that the pH at a distance of 4 m from the discharge point should not be lower than 6.5, and that the scrubber should not take up more than 12% of the emitted NO_X_. Limits are also proposed for turbidity and polycyclic aromatic hydrocarbons in phenanthrene equivalents.

The simplest (and cheapest) option is an open-loop scrubber, which discharges the resulting acidified water back to the sea surface. An alternative approach uses closed-loop scrubbers that limit the acidic discharge by neutralising the absorbed acid and recycling part of the treated water. The description “closed-loop” is somewhat misleading since these scrubbers discharge recycled neutralised water back to the sea surface, but in much smaller volumes that open-loop scrubbers.

The aim of the work reported here is to assess the potential consequences for the Baltic Sea of extensive use of scrubber technology in the coming decades.

## Materials and Methods

### Emission scenarios

We have developed five future scenarios that differ with respect to the sulphur content of the fuel, and also the percentage of the fleet using open-loop wet scrubbers, i.e., scrubbers that discharge their effluent back to the surface water (Table 1; see also Claremar et al. (2017). We have not attempted to model the use of closed-loop scrubbers, whose effluent is dependent on scrubber design: widespread use of closed-loop scrubbers would correspond to a scenario within range of the extreme scenarios used here. Scenario no. 1 corresponds to the fuel content regulation up to December 2014, and scenario no. 3 to the regulation from January 2015. Scenario no. 2 has been included since it was proposed in Sweden as a low-cost alternative to the 0.1% SECA regulation that came into force in January 2015. Scenarios no. 4 and 5 explore worst case scenarios for the use of wet scrubbers. Since the economics of scrubbers are based on low-cost (i.e., high-sulphur) fuel, it is assumed that the fuel used will have an average sulphur content of 2.7%, corresponding to the current average outside SECA (ENTEC 2005). The current guidelines for scrubber water discharge state that the seawater pH at 4 m from the discharge point should not be less than 6.5 (HELCOM 2016). Since this should be achievable without neutralisation of the open-loop scrubber effluent, we assume that the strong acid generated from SO_X_ is discharged to the surface water. Since regulation of NO_X_ emissions is gradual and at an early stage, we have assumed that these emissions will increase at the same rate as the shipping traffic. This is assumed to increase in accordance with the TREMOVE European transport model (De Ceuster et al. 2006), which gives an increase of 2.5% per year for cargo traffic and 3.9% per year for passenger traffic.

**Table 1:** Scenarios investigated for the sulphur content of marine fuels and for the use of wet scrubbers

Scenario no.	Shipping not using wet scrubbers		Shipping using wet scrubbers^b^	
	% of total	% sulphur in fuel^a^	% of total	% sulphur in fuel
1	100	1.0	0	
2	100	0.5	0	
3	100	0.1	0	
4	50	0.1	50	2.7
5	0		100	2.7

^a^SECA regulations changed from 1 to 0.1% in January 2015

^b^It is assumed that 96% of the sulphur is taken up in the scrubber (Kjølholt et al. 2012) and that the scrubber water is discharged untreated

For each scenario, three model runs were carried out. The first was a control run without emissions of SO_X_ and NO_X_, although the nitrate content of the surface water was increased to correspond to the amount of anthropogenic NO_X_ deposition: this was done to ensure that the effects of acidification could be examined in isolation. The second run included SO_X_ and NO_X_ deposition from shipping alone; and the third run included deposition from both shipping and terrestrial sources.

### Atmospheric modelling

The spatial distribution of atmospheric deposition of SO_X_ and NO_X_ from the ships is estimated by the atmospheric chemical transport model EMEP (Simpson et al. 2012). The model was run for the meteorological years 2009–2011 and the deposition fields from these 3 years were scaled with ship emission scenarios covering other periods. Details of the method used to determine the atmospheric deposition are described in Claremar et al. (2013) and Omstedt et al. (2015). Historical data were produced by a combination of emission databases and deposition from the EMEP model (Omstedt et al. 2015). Atmospheric background concentrations for future scenarios follow the RCP 4.5 emissions scenarios from 2010 (Lamarque et al. 2010) and deposition simulations (Engardt and Langner 2013) using the MATCH model (Robertson et al. 1999). For the ship contributions to atmospheric deposition, the RCP 4.5 information (Eyring et al. 2010) is replaced by the different traffic and scrubber scenarios described in the previous section and in Table 1.

### Biogeochemical modelling

**Fig. 1:**
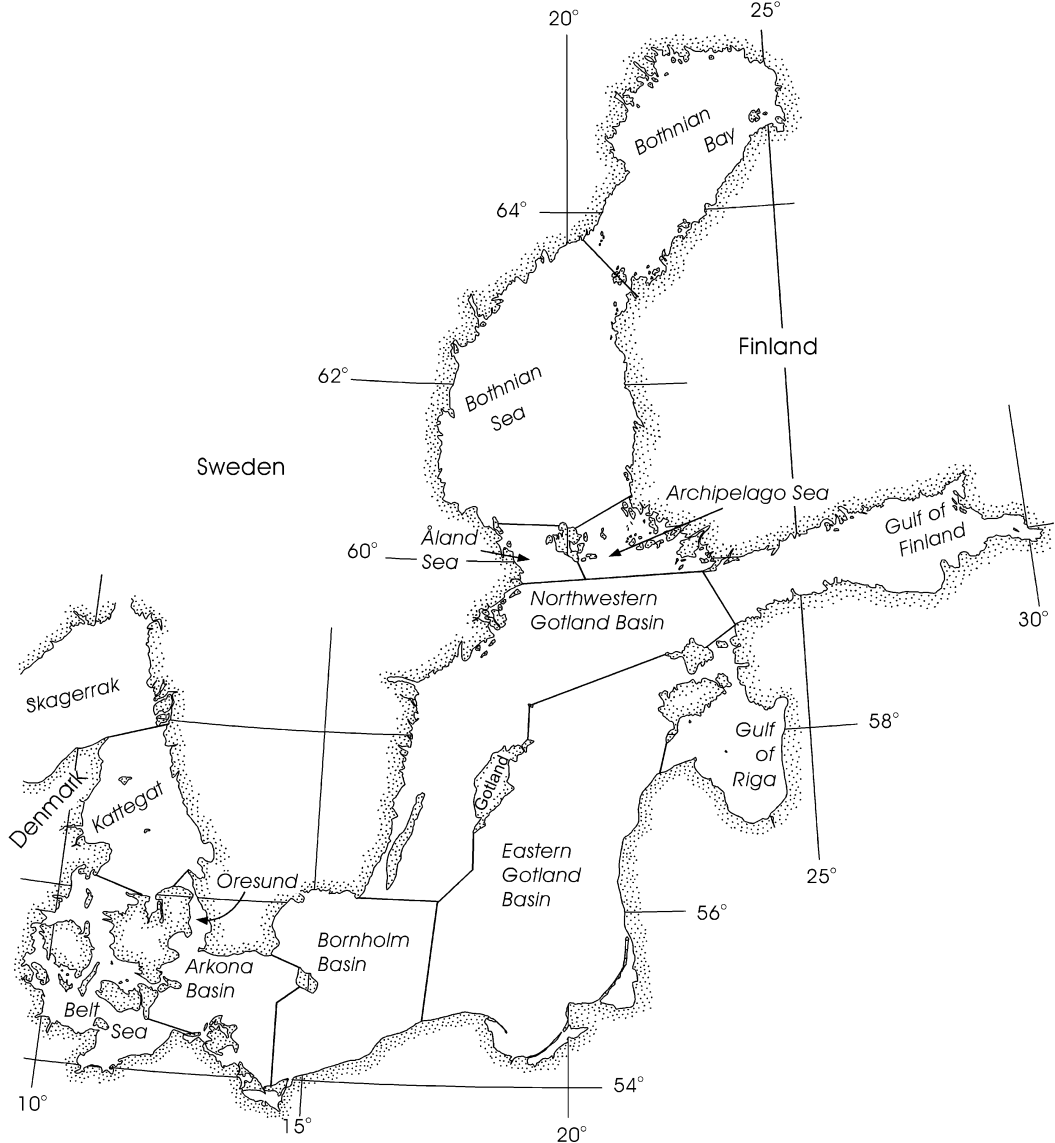
Map of the Baltic Sea showing the 13 sub-basins modelled in this work

Climate development follows the IPCC Special Report on Emission Scenarios (SRES) A1B projection (Nakicenovic and Swart 2000). Using the approach of Omstedt et al. (2012), one main GCM scenario was considered for modelling the future changes (scenario 23). The scenario simulations were based on meteorological forcing fields from the ECHAM A1B GCM simulation down scaled by the regional climate model RCA3. RCA3 is a 3D dynamic regional model covering northern Europe with a grid of 50 by 50 km (Kjellström et al. 2005). The river run off and river loads of nutrients were modelled using land use change consistent with the A1B story line. The scenario predicts changes due to warming, increased carbon dioxide concentrations in the atmosphere, increased river runoff including increased nutrients and carbon loads. The potential inconsistency of using A1B scenario for meteorological data and RCP 4.5 scenario for atmospheric background concentrations is not expected to influence the results (temperature projection of RCP4.5 is actually more compatible with B1 scenario). The biogeochemical component of the model includes the cycles of carbon (organic and inorganic), nitrogen and phosphorus, where the organic carbon part includes three phytoplankton groups and one zooplankton group. The ship emission scenarios described in Table 1 were added to this climate scenario. The model is based on that of Omstedt et al. (2012), which divides the Baltic Sea into 13 basins (Fig. 1). For this work, the modelling set-up has been modified to better suit the demands of this study. The coastal buffer volumes previously used have been removed to improve the accuracy of the alkalinity loss due to acidic depositions. These buffer volumes mixed the river discharge with ocean water as it was added to the Baltic Sea model, which gave a better representation of the Baltic Sea physics. However, inclusion of the buffer volumes precludes the calculation of mass balances, which was one of the objectives of this work. Although the removal of the buffer volumes limits the accuracy of the calculated concentrations, the effect on the differences between simulations, which this work focuses on, will be relatively small.

## Results

### Projected pH and alkalinity changes due to shipping

In all figures, the projected pH and alkalinity changes are plotted as the difference between the individual scenarios and the corresponding control run where no emissions of SO_X_ or NO_X_ are included. In this way, the effect on the water chemistry of the emissions in an individual scenario can be examined.

**Fig. 2:**
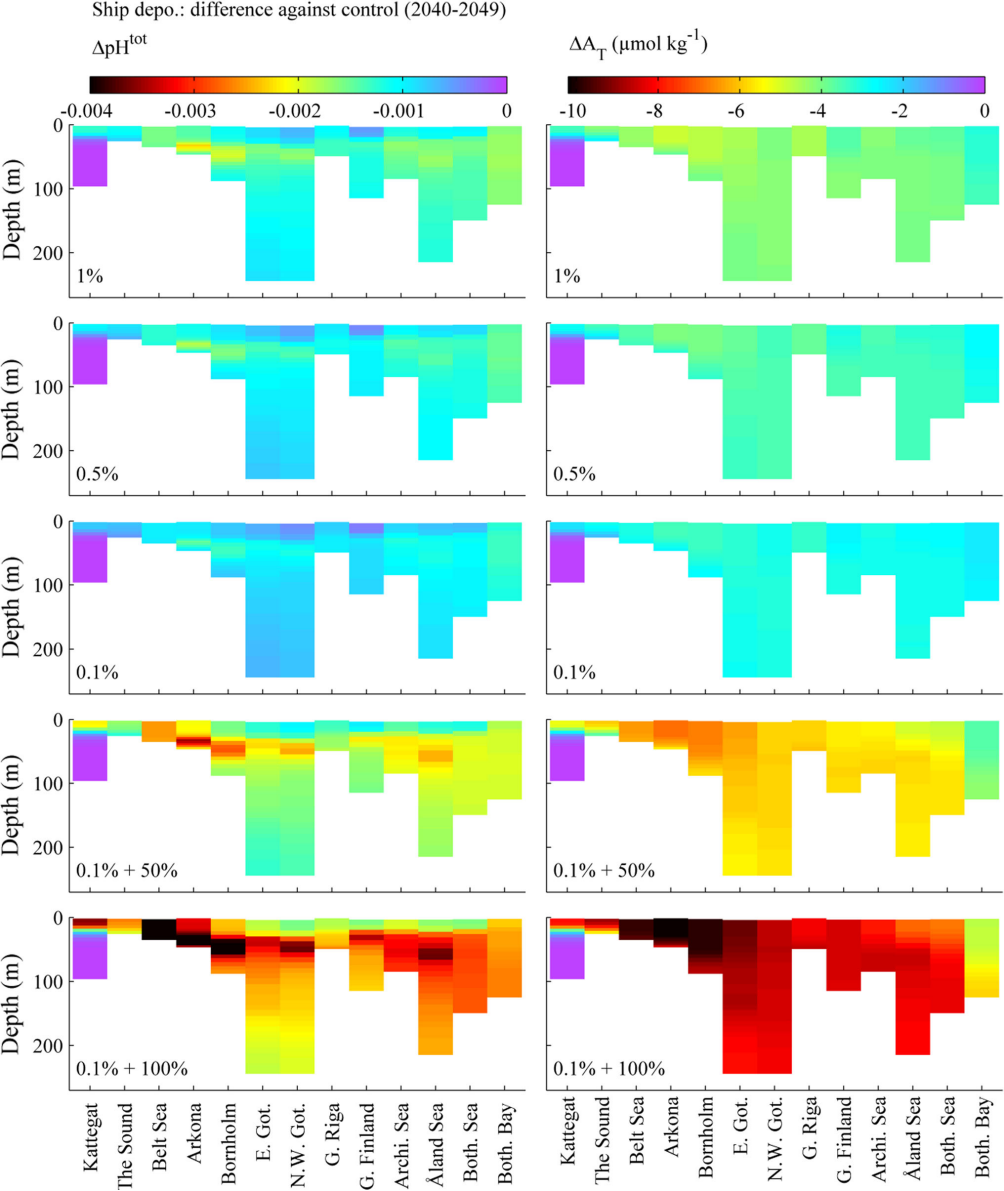
Transects from Kattegat to the Bothnian Bay for the five emission scenarios (Table 1), showing the pH and alkalinity changes due shipping alone

An overview of the projected consequences of shipping emissions in the 2040’s for all 13 basins of the Baltic Sea is shown in Fig. 2. It is clear that while the reduction from 1 to 0.1% sulphur introduced in January 2015 will have a relatively small effect on the chemistry of the marine environment, the introduction of scrubbers releasing untreated water can cause significant reductions in both pH and alkalinity. The effects are largest in the shallow and heavily trafficked areas around Denmark and the south of Sweden. Although the initial effect is confined to the surface mixed layer, the estuarine circulation of the Baltic Sea transports some of the additional acidification to deeper water.

**Fig. 3:**
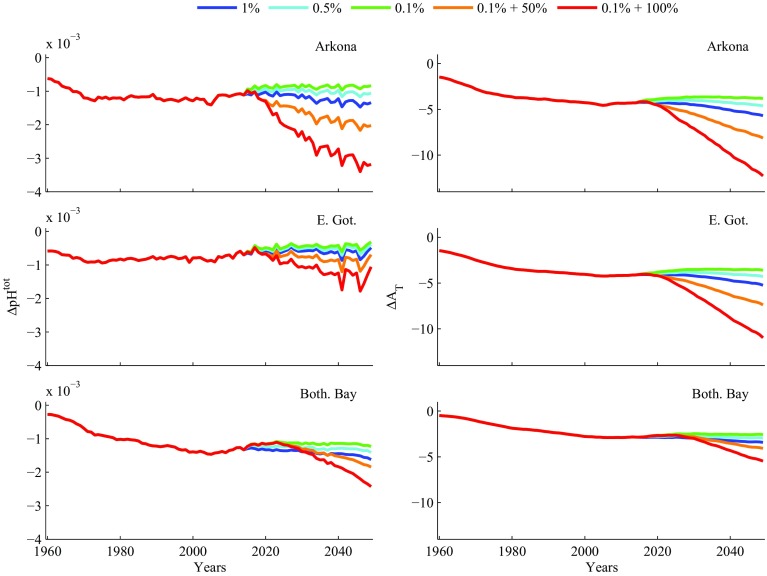
Modelled future changes in pH and alkalinity due to shipping alone, plotted as differences from the relevant control runs, in surface waters of the Arkona Basin, East Gotland Basin and the Bothnian Bay, according to the five scenarios described in Table 1

Figure 3 shows the development of surface water pH and alkalinity in three basins representing the heavily trafficked area at the entrance to the Baltic Sea (Arkona Basin); the Baltic Proper (East Gotland basin); and the northern Baltic (Bothnian Bay). Once again, reductions in the sulphur content of marine fuel below 1% have a relatively minor effect on the marine environment. However, discharge of non-neutralised scrubber waters can substantially increase the acidification effect. Two factors contribute to this difference. The first is that cheap, high-sulphur fuel is an important element in the economics of scrubber operation. The second is that discharge of scrubber effluent provides a much more direct and effective transport of the SO_X_- and NO_X_-derived acidity to the surface water than release from the smokestack and subsequent deposition over a wider sea (and land) area. Scoping calculations show that a fleet where ca. 80% of the shipping uses fuel with 0.1% sulphur, and the remaining 20% uses open-loop scrubbers, results in a total strong acid input equal to the same fleet using fuel with 1% sulphur, which was the situation up to 2014.

**Fig. 4:**
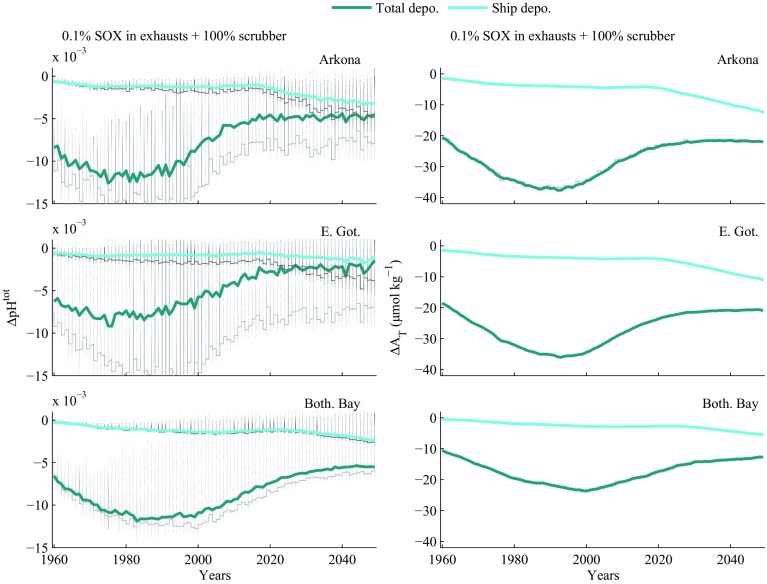
Modelled future changes in pH and alkalinity, plotted as differences from the relevant control runs, in surface waters of the Arkona Basin, East Gotland Basin and the Bothnian Bay, assuming that all ships use open-loop scrubbers (scenario 5). Total deposition includes both terrestrial and shipping sources. The grey vertical lines in the left panels show the annual range of pH changes

**Fig. 5:**
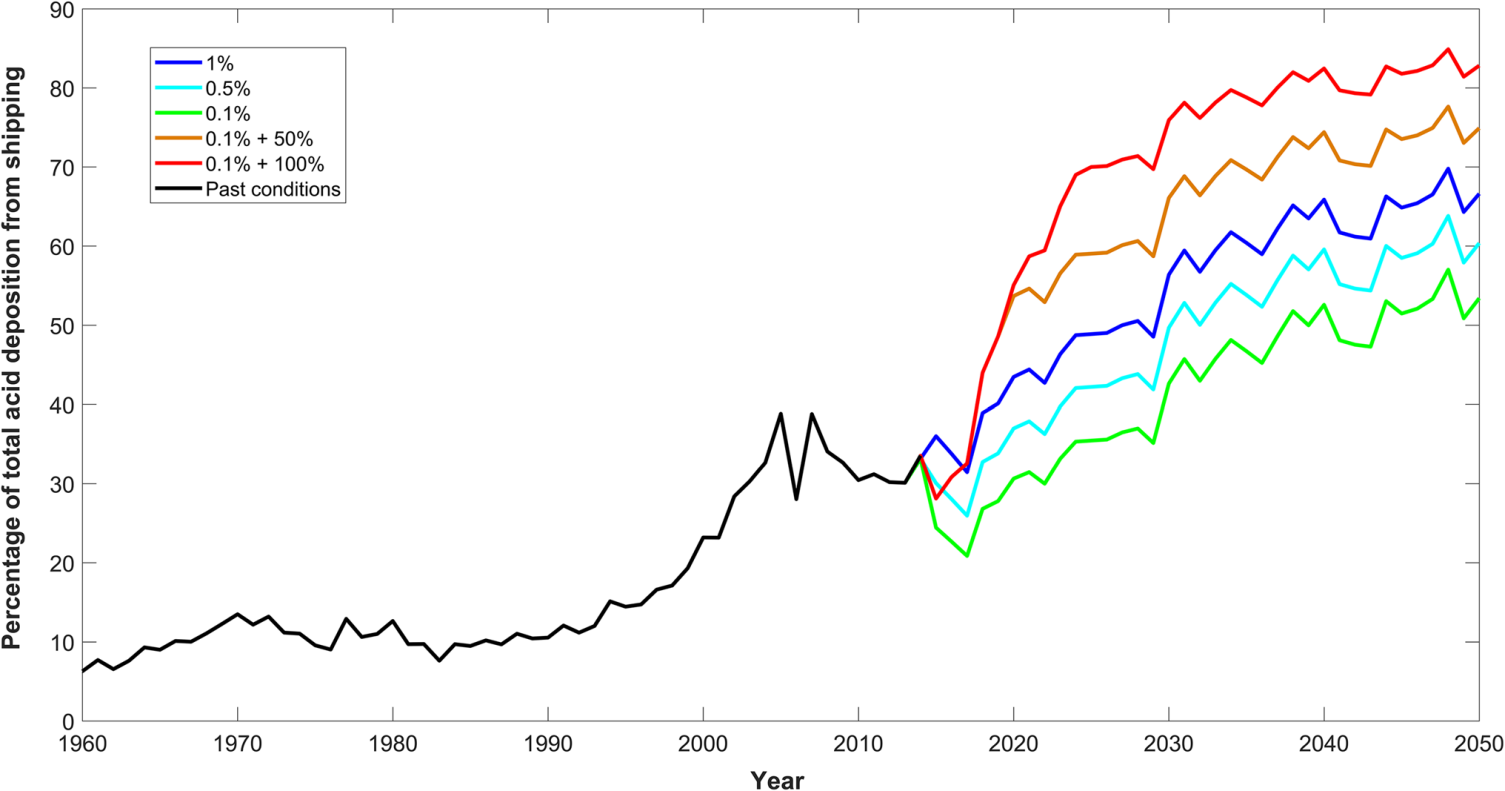
The proportion of strong acid deposition to the Baltic Sea due to shipping. The coloured lines show projections for the five scenarios presented in Table 1

Figure 4 compares the temporal development of shipping-derived acidification with the total (land plus shipping) in the same three basins. The reduction in land-derived acidification following the introduction of strict controls towards the end of the 20th century is seen very clearly, and together with increasing shipping traffic results in shipping contributing an ever larger proportion of acidification by SO_X_ and NO_X_. The figure also shows (in grey) the variability that underlies the yearly average values plotted. Figure 5 shows the percent contribution of shipping to the total deposition of acid predicted for the Baltic Sea according to the five emission scenarios. Although shipping emissions increase and eventually dominate the strong acid input under all scenarios (Fig. 5), terrestrial emissions still have the larger cumulative effect on Baltic Sea surface water (Fig. 4). Strong acid depositions thus have a relatively long-term effect on Baltic Sea chemistry.

### Acid deposition reduces CO_2_ uptake

While the oceans act as a sink for anthropogenic carbon dioxide, deposition of strong acids can be expected to result in a reduced CO_2_ uptake due to the lowering of both pH and alkalinity. We have used our model results to examine this effect, and find a clear linear relationship between strong acid input and reduced CO_2_ uptake on an annual basis. The molar ratio between reduced CO_2_ uptake and strong acid addition is 0.812 ± 0.001 for total emissions and 0.826 ± 0.006 for shipping emissions. This indicates that shipping in the Baltic Sea has an additional, indirect “carbon footprint” amounting to ca. 82% of strong acid emissions.

### To what extent do the chemical changes accumulate in the Baltic Sea?

**Fig. 6:**
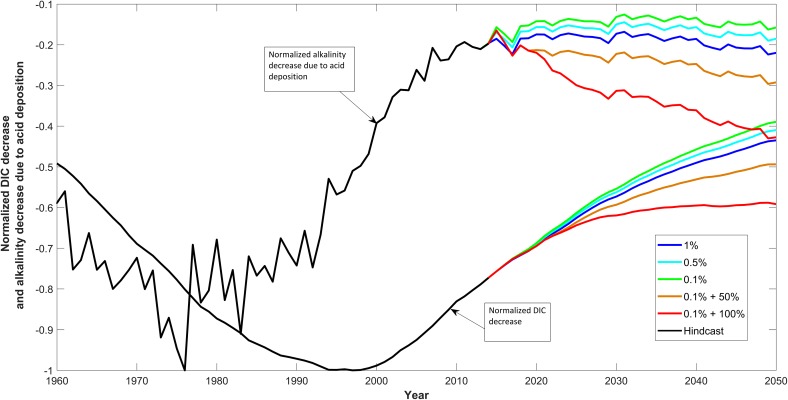
Decreases in the alkalinity content of the entire Baltic Sea due to strong acid deposition, together with the resulting calculated changes in dissolved inorganic carbon (DIC) compared to the relevant control runs. Both parameters are normalised to their maximum decrease

Our modelling projections show that the deposition of strong acids to the Baltic Sea reduces pH and alkalinity to a limited extent (Fig. 2), and also results in a reduced uptake of CO_2_ and therefore a reduced total carbonate concentration. An important question to be addressed is whether these effects are transient due to export to the North Sea via the Skagerak, or whether they accumulate in the Baltic Sea in the longer term. A clear indication is provided by Fig. 6, which compares the temporal development of the total acid deposition with the resulting change in total carbonate concentration. The acid deposition peaked in the period 1970–1980 as increasingly effective measures were taken to reduce terrestrial emissions, while the reduction in total carbonate concentration peaked some 15–20 years later. These results indicate that the effect of the emissions peak was indeed transient. However, it can also be seen from Fig. 6 that increasing shipping emissions are projected to cause a second peak in total strong acid deposition in the coming decades if wet scrubber technology is widely used. In this case, the changes in dissolved inorganic carbon are projected to level off.

## Discussion

This study has focused on the modelled acidification of the Baltic Sea resulting from the release of sulphur and nitrogen oxides from smokestacks and/or untreated scrubber effluent. This represents a significant advance on previous studies of acidification by SO_X_ and NO_X_ that have modelled larger areas on an annual basis (Hunter et al. 2011), or have examined the temporal development in small ocean areas while neglecting lateral transport (Hassellöv et al. 2013; Hagens et al. 2014). However, this first assessment for the Baltic Sea does not provide a complete picture of the potential consequences of wet scrubber operation for three reasons.

- Firstly, shipping activities are not evenly distributed over the surface water of the different basins, but in many cases confined to specified shipping routes. This means that discharges of scrubber effluent will be concentrated along the shipping routes before spreading to the remainder of the basin by lateral advection. This effect could not be explored in the basin-oriented model described here. Stips et al. (2016), using a 10 km horizontal resolution, modelled the effects of SO_X_ deposition in the North Sea over a 1-year period, concluded that the acidification effects are largely confined to coastal areas close to major ports. Examining the consequences of scrubber operation along the major shipping routes would, however, demand a model with a significantly smaller horizontal grid size.
- Secondly, the strong acids are not the only significant chemical component of scrubber effluent: toxic substances in the form of heavy metals, organic compounds and particulate matter have also been shown to be present in scrubber effluents. These components can be expected to have different chemistries for wet scrubber input and atmospheric deposition, since in the latter case, the material will be subject to chemical transformation in the atmosphere before deposition (Russell et al. 1999).
- Thirdly, smokestack gases and scrubber effluents also contain plant nutrients, notably nitrogen and iron. Further work is needed on the consequences for the marine environment of the toxic and nutrient components of smokestack gases and scrubber effluent. This is particularly important in the case of scrubber effluent, since the technology is developing rapidly, and the currently available analytical data on scrubber effluents are restricted to first generation scrubbers that may not be representative of the current generation.

In contrast to other estimates, the changes in pH on a basin scale are relatively small, being largest in the Arkona Basin. However, basin scale modelling does not allow us to see the smaller scale effects e.g., close to harbours, which are highlighted in the higher-resolution model of Stips et al. (2016). Figure 3 shows pH reductions due to the most extreme scenario of shipping emissions (red lines) ranging between 0.001 and 0.003 over a period of approximately 30 years, giving a maximum change of the order of 0.0001 pH unit per year. This is the annual rate estimated by Hunter et al. (2011) for the Baltic Sea on a “business as usual” basis, corresponding to the blue lines in Fig. 3, where the annual rate of reduction is an order of magnitude lower than the extreme scenario (red lines). Our new estimates are therefore significantly lower, which may in part be due to the ability of the Baltic Sea to export these chemical changes to the North Sea (Fig. 6). This export arises since, in contrast to most other contaminants, strong acids are not exported to deep water via the marine food chain and thus do not accumulate there to a large extent. Thus the deep water alkalinity changes in Fig. 2 are relatively modest in comparison with the surface waters of the Bornholm and Arkona Basins and the Belt Seas that contribute to the surface outflow from the Baltic Sea to the Kattegat, Skagerak and North Sea. However, the other contaminants associated with smokestack emissions and scrubber water discharge (organic compounds, trace metals and small particles) can be expected to enter the food chain and accumulate in the Baltic deep waters, thus affecting Baltic Sea chemistry over much longer time periods that the strong acid input. There is thus a need for further research that focuses on quantifying the emissions of these contaminants from smokestacks and scrubbers, and examining their fate in the Baltic Sea.

The results presented here focus on the effects of SO_X_ and NO_X_ ship emissions in relation to a control scenario where no such emissions occur. The control scenario is by no means constant, but reflects the climate-driven changes expected according to the A1B storyline and changes in deposition of emissions from other sources following RCP4.5. Müller et al. (2016) have recently reviewed the temporal development of alkalinity in the Baltic Sea, and have shown that the alkalinity is currently increasing. This may be connected to the observed increase in organic carbon concentrations (Fleming-Lehtinen et al. 2015), since it has been shown that organic matter contributes to the total alkalinity in the Baltic Sea (Kulinski et al. 2014). The alkalinity reduction due to the deposition of acidic oxides thus occurs against the background of increasing alkalinity that is included in our control runs.

## Conclusions

Basin scale modelling projections of the Baltic Sea through to 2050 indicate that shipping will become the major source of strong acid addition to surface waters, most particularly if there is widespread use of wet scrubber systems. These strong acid additions result in a reduced uptake of atmospheric carbon dioxide of approximately 82% on a molar basis. The effects on the chemistry of the Baltic Sea are projected to be transient with a timescale of 15–20 years. The overall consequences for the alkalinity and pH of the Baltic Sea are small on a basin scale.
